# Role of the human solute carrier family 14 member 1 gene in hypoxia-induced renal cell carcinoma occurrence and its enlightenment to cancer nursing

**DOI:** 10.1186/s12860-023-00473-6

**Published:** 2023-03-18

**Authors:** Jing Shi, Ruili Sha, Xilan Yang

**Affiliations:** Department of Urology, Nanjing First Hospital, Nanjing Medical University, Qinhuai District, 68 Changle Road, Nanjing, 210012 China

**Keywords:** Human Solute Carrier Family 14 Member 1, Hypoxia, Renal Cell Carcinoma, Nursing

## Abstract

**Background:**

Hypoxia is considered a critical contributor to renal cell carcinoma progression, including invasion and metastasis. However, the potential mechanisms by which it promotes invasion and metastasis have not yet been clarified. The purpose of this study was to investigate the role and mechanism of hypoxia-induced renal cell carcinoma and provide evidence-based medical proof for improvements to postoperative nursing of renal cell carcinoma patients. A total of 64 patients with renal cell carcinoma were divided into the observation group (nursing based on oxygen administration) and the control group (conventional nursing). Renal function indexes, serum inflammatory factors, and tumor markers were evaluated. The human renal cell carcinoma cell line A498 under hypoxia/normoxia was used as an experimental model in vitro and the biological characteristics and mitochondrial function of the cells were assessed.

**Results:**

Nursing based on oxygen administration decreased the value of renal function indexes, serum inflammatory factors, and tumor markers in renal cell carcinoma patients. Hypoxia was found to induce A498 cell invasion, migration, and the release of inflammatory cytokines, while repressing human solute carrier family 14 member 1 gene expression. Elevated levels of solute carrier family 14 member 1 expression induced mitochondrial reactive oxygen species accumulation, diminished the intracellular adenosine triphosphate level, and destroyed both mitochondrial membrane potential integrity and mitochondrial morphology. Overexpression of the solute carrier family 14 member 1 gene could abolish hypoxia-induced invasion, reduce the migration of A498 cells, inhibit the hypoxia-induced release of inflammatory cytokines, and arrest the cell cycle at the G1/S checkpoint.

**Conclusions:**

These data reveal that nursing based on oxygen administration can improve the clinical efficacy of renal cell carcinoma therapies, being safe and effective. The results elucidate a mechanism wherein the solute carrier family 14 member 1 gene participates in the occurrence and development of hypoxia-induced renal cell carcinoma in a mitochondria-dependent manner.

**Supplementary Information:**

The online version contains supplementary material available at 10.1186/s12860-023-00473-6.

## Background

Renal cell carcinoma (RCC) originates from either the cancerous renal cortex or renal tubular epithelial cells, and is the second most lethal urological malignancy, accounting for 90–95% of kidney neoplasms. Its incidence and mortality are steadily increasing at a rate of approximately 2–3% every decade [1, 2]. There are three main histological subtypes of RCC, including clear cell RCC (ccRCC), papillary RCC, and chromophobe RCC, which jointly represent over 85% of all primary renal malignancies [3]. The treatment for renal parenchymal tumors has changed over time, and this trend continues today as a result of technological progress made with clinical research and improved diagnostic and therapeutic tools. Unfortunately, the traditional treatment methods of chemotherapy and radiotherapy have proven to be ineffective and lead to multidrug resistance in target cells. Although modern approaches were initially popular, such as immunotherapy and the use of angiogenesis inhibitors, subsequent studies have shown their limited and confounding effects. Radical nephrectomy and nephron-sparing surgery are still the only effective treatments for localized RCC, but the characteristics of RCC recurrence and metastasis lead to a 5-year survival rate of approximately 12%, posing a great challenge to clinical therapies [4]. Therefore, the development of a new and accurate therapeutic strategy is needed to improve the clinical treatment of RCC. Importantly, it must overcome the issue of RCC’s poor response to chemotherapy, radiotherapy, and immunotherapy.

Adaptation to hypoxia (≤ 2% O_2_) is involved in many physiological and pathological conditions, such as stroke, tissue ischemia, and cancer [5]. A recent report indicated that long-term hypoxia is a major driving force of cancer tumorigenesis [6]. Hypoxia is an indicator of poor prognosis in a variety of solid tumors, including prostate [7], cervical [8], breast [9], and head and neck cancers [10]. Hypoxia participates in epithelial-to-mesenchymal transition (EMT), angiogenesis (vascular endothelial growth factor (VEGF)), the shift from oxidative phosphorylation to anaerobic glycolysis and energy metabolism (glucose transporter isoform 1 (GLUT1)), the promotion of proliferation, the induction of invasion and metastasis, and the inhibition of cancer cell apoptosis, ultimately leading to carcinogenesis [11, 12]. In this case, the manipulation of the hypoxic microenvironment can be considered a means to prevent or reverse malignant transformation. Mitochondrial dysfunction is an important factor that plays an active role in the hypoxia response by regulating tumor cell proliferation, apoptosis, neovascularization, invasion, and metastasis [13]. It is therefore imperative that normal cells remove this hypoxia-induced mitochondrial damage. However, the exact mechanism by which hypoxia-induced mitochondrial dysfunction contributes to tumor occurrence and development only attracted interest a few years ago, and still remains unclear. It is known that hypoxia is a typical microenvironment characteristic of almost all solid tumors. RCC derives from highly heterogeneous renal tubular epithelial cells and is highly sensitive to hypoxia. The hypoxic tumor environment is highly prevalent in poorly vascularized and necrotic RCC tumor cores [14]. It has been reported that the hypoxia-inducible transcription factor (HIF) signaling pathway plays a critical role in the genetic instability and pathogenesis of RCC [15]. At present, therapeutic drugs targeting HIF, VEGF, and phosphatidylinositol 3-kinase (PI3K)/mammalian target of rapamycin (mTOR) are important first-line treatment options for advanced RCC patients, but only a small proportion of patients benefit from them [16]. These reports prompted us to investigate the molecular pathways underpinning RCC tumorigenesis to identify more effective therapeutic targets in hypoxia-induced RCC.

The hypoxia regulates numerous target genes associated with malignant behavior, including angiogenesis, cell survival, proliferation, mitochondrial function, and metabolism. Mitochondrial function is involved in the initiation and progression of malignant tumors and modulates multiple processes, such as altering glucose metabolism, destroying redox balance, producing reactive oxygen species (ROS), and damaging the intrinsic apoptotic pathway, which plays a central role in the growth, metastasis, and tumorigenesis of RCC [17, 18]. Urea transporter B (UT‐B), which is encoded by the *SLC14A1* (human solute carrier family 14 member 1) gene, has been verified to be widely distributed in numerous tissues, including the kidney, brain, heart, liver, and other tissues; it regulates the urea concentration, blood pressure, bone metabolism, and cardiac functions [19]. A deficiency in *SLC14A1* gene expression or its dysfunction contributes to the pathogenesis of diseases. For example, *SLC14A1* gene knockout mice were reported to exhibit mitochondrial dysfunction and increased rates of apoptosis of the heart [20]. The expression of the *SLC14A1* gene was also found to induce melanoma B16 cell death via the activation of p53 and the induction of mitochondrial apoptosis [21]. Therefore, a better understanding of the mechanisms of *SLC14A1* in malignancies may lead to the development of more effective and more specific drugs. The primary objective of this study was to investigate the role and mechanism of hypoxia-induced RCC, as well as to provide evidence-based medical proof for ways to improve postoperative nursing in the context of this disease. We also explored the effect of the *SLC14A1* gene on mitochondrial function in a human RCC cell line and elucidated that it participates in the occurrence and development of hypoxia-induced RCC in a mitochondria-dependent manner. In summary, the findings suggest that *SLC14A1* gene expression sensitively responds to hypoxia, and hence serves as an ideal prospective target for RCC treatment.

## Results

### Effect of normoxic/hypoxic conditions on the biological characteristics of A498 cells

**Fig. 1:**
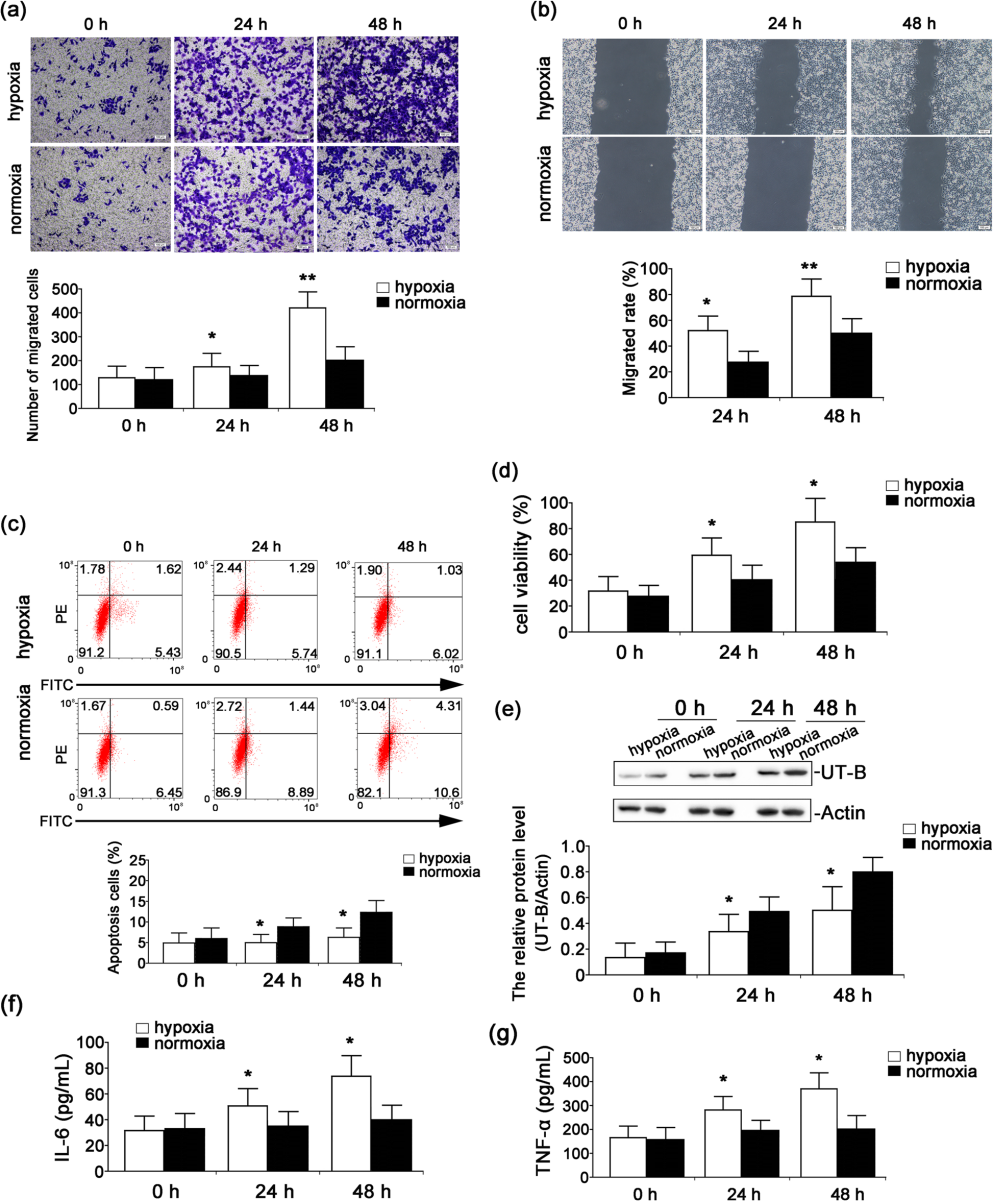
Effect of hypoxia/normoxia on the biological characteristics of A498 cells. A498 cells were cultured at 37 °C in a humidified incubator with 5% CO_2_, 2% O_2_, and 93% N_2_ (hypoxia) or with 5% CO_2_ and 95% O_2_ (normoxia) for 24 and 48 h. **a** The invasion of A498 cells was detected by the transwell assay. **b** The migration of A498 cells was examined by the wound healing assay. **c** The apoptosis of A498 cells was measured by flow cytometry. **d** The viability of A498 cells was assessed by the CCK-8 assay. **e** Expression of the UT-B protein was detected by western blot. Full-length blots/gels are presented in Supplementary file 5. The levels of IL-6 (**f)** and TNF-α (**g**) in the culture supernatant were determined by a Beckman Coulter AU5800 automatic biochemical analyzer. Each bar represents the median expression obtained from 3 independent experiments. *P < 0.05, **P < 0.01 vs the corresponding normoxic group

To investigate the effect of hypoxia on the biological characteristics of RCC, A498 cells were cultured under normoxic/hypoxic conditions. The results show that hypoxia induced the invasion and migration of A498 cells in a time-dependent manner when compared to the normoxic cells (Fig. 1a-b). Flow cytometry analysis indicated that hypoxia inhibited A498 cell apoptosis compared to the normoxia (Fig. 1c). In addition, the viability of A498 cells was significantly increased with the extension of incubation time under hypoxia (Fig. 1d). Western blot analysis revealed that UT-B expression in A498 cells cultured under hypoxia was markedly lower than that in the corresponding normoxic group (Fig. 1e). Moreover, hypoxia significantly increased the production of the serum inflammatory cytokines interleukin 6 (IL-6) and tumor necrosis factor α (TNF-α) compared to the normoxic group (Fig. 1f-g). Such tumor-related inflammatory factors promote the development and progression of cancer, so hypoxia is closely associated with changes in the biological characteristics of RCC.

### Effect of oxygen treatment on the clinical efficacy of RCC patients

It is known that hypoxia is a significant characteristic of RCC patients. Therefore, this study aimed to explore the effect of oxygen treatment on the clinical efficacy of RCC patients. The results show that the observation group had significantly decreased values for biochemical indexes compared to the control group, as indicated by decreased levels of the renal function indexes blood urea nitrogen (BUN) and creatinine (Cr), the serum inflammatory factors IL-6 and TNF-α, and the tumor markers carcinoembryonic antigen (CEA) and carbohydrate antigen 50 (CA50) (*t* = 2.213, 2.490, 2.035, 2.500, 2.281, and 2.282, respectively, all *P* < 0.05, Table 1). Moreover, the observation group had fewer postoperative complications (total incidence) and a lower incidence of RCC metastasis and recurrence compared to the control group (*t* = 4.267, 5.851, and 4.267, respectively, all *P* < 0.05, Table 2). The present study also demonstrated that the intervention of oxygen treatment significantly alleviated the anxiety and depression of RCC patients, while nursing satisfaction was also significantly improved compared to the corresponding control group (*t* = 6.478, 5.107, and 3.925, respectively, all *P* < 0.05, Table 3).

**Table 1:** Comparison of clinical index ($\overline{x }$  ±  s)

Groups	n	BUN (mmol/L)	Cr (μmol/L)	IL-6 (pg/mL)	TNF-α (pg/mL)	CEA (μg/L)	CA50 (U/mL)
Observation group	32	10.62 ± 1.49^*^	112.47 ± 7.33^*^	6.25 ± 0.67^*^	52.13 ± 6.23^*^	4.26 ± 0.85^*^	14.75 ± 0.86^*^
Control group	32	11.53 ± 1.78	119.97 ± 15.38	6.57 ± 0.63	56.31 ± 7.11	4.81 ± 1.07	15.4 ± 1.39
*t*	-	2.213	2.490	2.035	2.500	2.281	2.282
*P*	-	0.031	0.015	0.046	0.015	0.026	0.026

Note: *, *P* < 0.05 *vs* control group

**Table 2:** Comparison of postoperative complications, metastasis and recurrence between the two groups (%)

Groups	n	Incision infection	Bleeding	Urinary tract infection	Total incidence	Recurrence	Metastasis
Observation group	32	1(3.10)	1(3.10)	2(6.30)	4(12.5)*	3(9.40)*	2(6.30)*
Control group	32	3(9.40)	4(12.5)	4(12.5)	11(34.4)	11(34.4)	8(25.0)
*X*^*2*^	-	1.067	1.953	0.736	4.267	5.851	4.267
*P*	-	0.302	0.162	0.391	0.039	0.016	0.039

Note: *, *P* < 0.05 *vs* control group

**Table 3:** Comparison of incidence of anxiety and depression and satisfaction between the two groups (%)

Groups	n	anxiety	depression	Treatment satisfaction
Observation group	32	8 (25.0)*	10 (31.3)*	27 (84.4)*
Control group	32	18 (56.3)	19 (59.4)	20 (62.5)
*X*^*2*^	-	6.478	5.107	3.925
*P*	-	0.011	0.024	0.048

Note: *, *P* < 0.05 *vs* control group

### Characteristics of the *SLC14A1* gene in hypoxia-associated RCC determined by gene expression profiling interactive analysis (GEPIA)

**Fig. 2:**
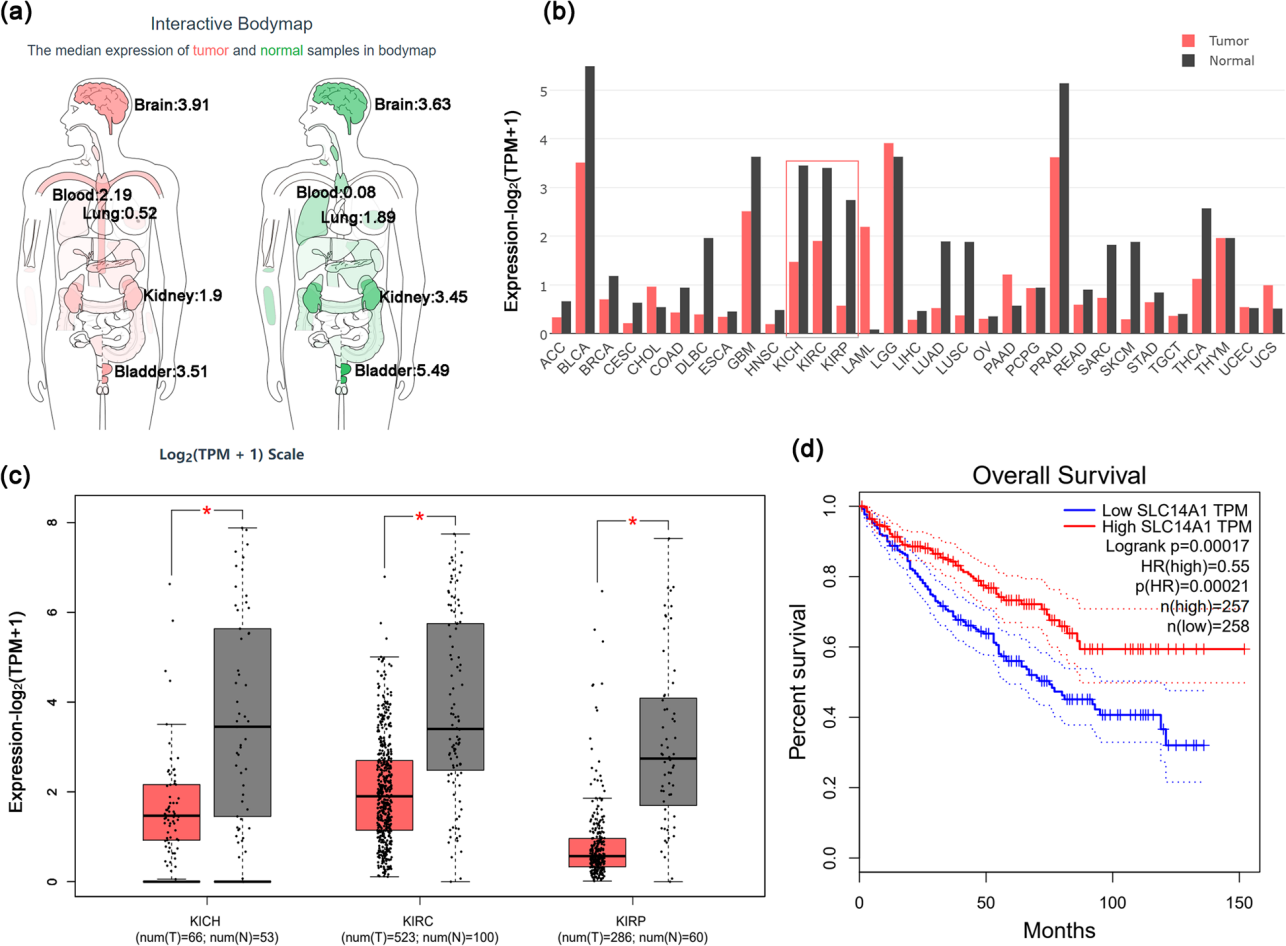
Expression characteristics of the *SLC14A1* gene in hypoxia-associated RCC using Gene Expression Profiling Interactive Analysis (GEPIA). **a** The median expression of tumor (red) and normal (blue) samples in the bodymap. **b** The gene expression profile across all tumor samples and paired normal tissues (bar plot). The height of each bar represents the median expression of a certain tumor type or normal tissue. KICH: kidney chromophobe; KIRC: kidney renal clear cell carcinoma; KIRP: kidney renal papillary cell carcinoma; **c** *SLC14A1* gene expression in box plot, including 66 cases of KICH with 53 paired normal tissues, 523 cases of KIRC with 100 paired normal tissues, and 286 cases of KIRP with 60 paired of normal tissues. Data were obtained from the Genotype-Tissue Expression (GTEx) project and are presented as the mean ± quartile values. *P < 0.05 by the unpaired *t*-test. **d** Relationship of *SLC14A1* gene expression with the overall survival of 515 RCC patients, as obtained by the online Kaplan–Meier survival plotter

An interactive bodymap shows that the *SLC14A1* gene is widely distributed in human tissues and organs, such as the brain, lungs, kidneys, and bladder. It is particularly highly expressed in the human urinary system, especially in the kidney and bladder. Moreover, Fig. 2a shows that *SLC14A1* gene expression in RCC tissue (red) is lower than that in the corresponding normal renal tissue (blue). Figure 2b illustrates its gene expression profile across all tumor samples and paired normal tissues, revealing that *SLC14A1* gene expression levels in kidney chromophobe (KICH), kidney renal clear cell carcinoma (KIRC), and kidney renal papillary cell carcinoma (KIRP) tissues (red) are significantly lower than that in normal renal tissue (brown, Fig. 2b). Box plots show that *SLC14A1* gene expression is significantly relatively lower in KICH, KIRC, and KIRP tissues (Fig. 2c). The relationship of *SLC14A1* gene expression with the overall survival of RCC was analyzed using the Kaplan–Meier plotter. High *SLC14A1* gene expression was found to prolong overall survival to some extent in patients with RCC (Logrank *P* = 0.00017, Fig. 2d). Moreover, Kaplan–Meier plotting demonstrated that the hazard ratio (HR) of the *SLC14A1* gene was 0.55 (where HR < 1 is identified as a protective factor for disease), thereby supporting it as an important protective factor for RCC [*P*(HR) = 0.00021].

### *SLC14A1* gene expression induced mitochondrial dysfunction in A498 cells

**Fig. 3:**
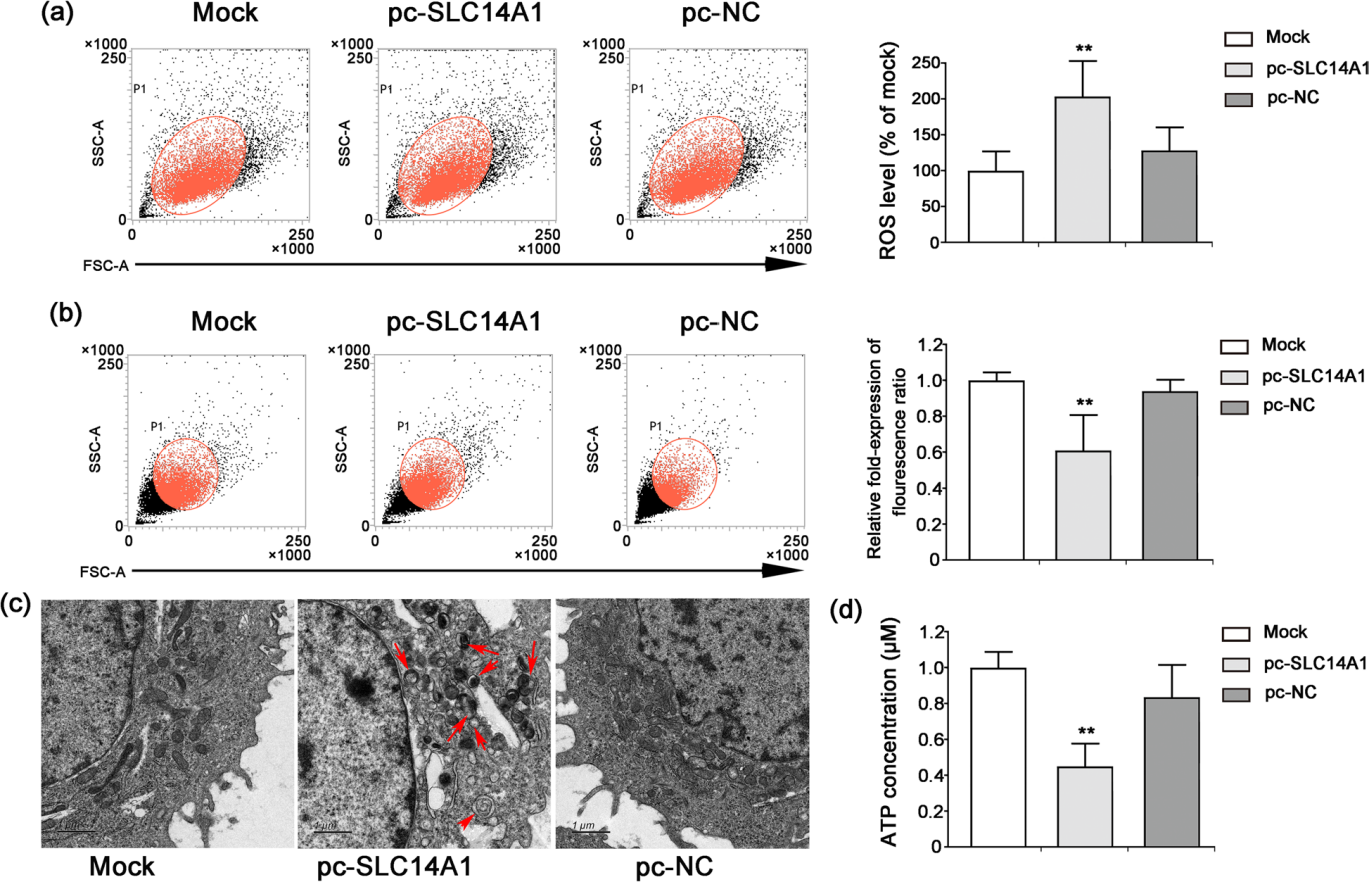
Effect of *SLC14A1* overexpression on mitochondrial dysfunction in A498 cells. A498 cells were transfected with either the pc-*SLC14A1* vector (2.5 μg/ml) or pcDNA3.1 null vector (pc-NC, 2.5 μg/ml) for 48 h. **a** The fluorescent probe MitoSOX™ Red was used to assess mitochondrial ROS level. **b** The JC-1 probe was used to determine changes in the mitochondrial membrane potential. **c** Observation of A498 cell morphology was performed using electron microscopy (× 5,200). Red arrows indicate abnormal mitochondria structure, including mitochondrial swelling and rupture. Scale bar = 1 μm. **d** The ATP level of A498 cells was evaluated using a luciferase-based assay kit. Results are presented as the mean ± SD from 3 independent experiments. **P < 0.01 vs the pc-NC group

This study aimed to explore the effect of increased *SLC14A1* gene expression on mitochondrial function in A498 cells. As shown in Fig. 3a and b, treatment with the overexpression plasmid pc-*SLC14A1* significantly increased mitochondrial ROS generation and destroyed mitochondrial membrane potential (*ΔΨm*) integrity compared to treatment with the empty plasmid (pc-NC) (*P* < 0.01). Meanwhile, observations of representative mitochondrial morphology indicated that overexpression of the *SLC14A1* gene (pc-*SLC14A1*) caused the mitochondria to become swollen and vacuolated with cristae breakage and disappearance (Fig. 3c). Additionally, the pc-*SLC14A1* treatment significantly decreased the intracellular adenosine triphosphate (ATP) level relative to that of the pc-NC group (Fig. 3d).

### Effect of *SLC14A1* gene expression on hypoxia-induced biological characteristics of A498 cells

**Fig. 4:**
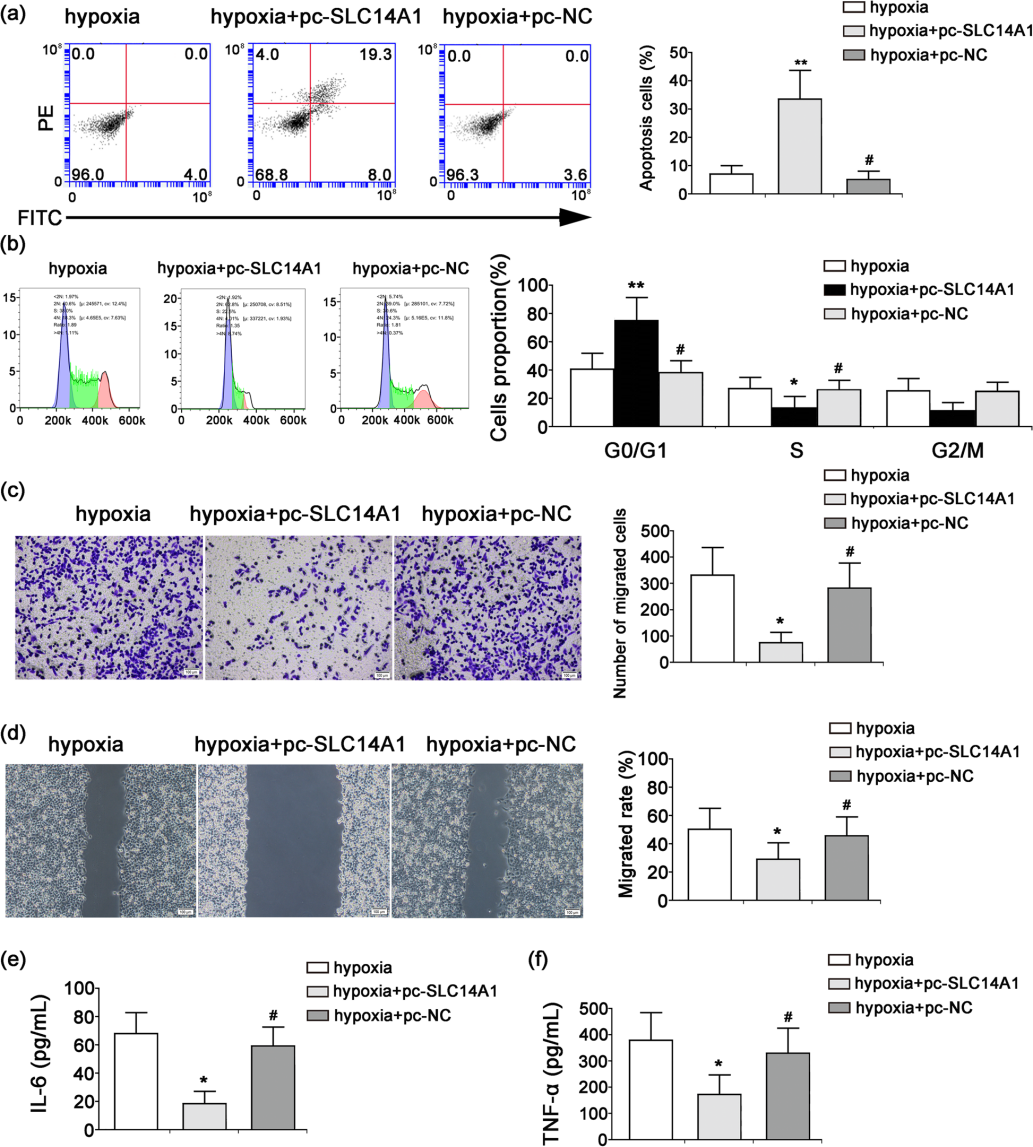
Effect of *SLC14A1* overexpression on hypoxia-induced biological characteristics of A498 cells. A498 cells were transfected with either the pc-*SLC14A1* vector (2.5 μg/ml) or pcDNA3.1 null vector (pc-NC, 2.5 μg/ml) and then cultured at 37 °C in a humidified incubator with 5% CO_2_, 2% O_2_, and 93% N_2_ (hypoxia) for 48 h. **a** The apoptosis of A498 cells was measured by flow cytometry. **b** The distribution of the cell cycle was calculated by flow cytometry. **(c)** The invasion of A498 cells was assessed by the transwell assay. **d** The migration of A498 cells was analyzed by the wound healing assay. The levels of IL-6 **e** and TNF-α **f** in the cell culture supernatant were measured by the Beckman Coulter AU5800 automatic biochemical analyzer. *n* = 3, **P < 0.01, *P < 0.05, ^#^*P* > 0.05 versus the hypoxia-alone group

To evaluate whether *SLC14A1* gene expression plays an important role in hypoxia-regulated apoptosis in A498 cells, the apoptotic level of cells was detected by annexin V- fluorescein isothiocyanate (FITC)/ propidium iodide (PI) staining. As shown in Fig. 4a, the hypoxia + pc-*SLC14A1* treatment significantly increased the number of apoptotic cells relative to the hypoxia-alone treatment (*P* < 0.05). There was no significant change in the number of apoptotic cells between the hypoxia-alone treatment and hypoxia + pc-NC treatment (*P* > 0.05). The cell cycle distribution was calculated by flow cytometry with PI staining. There was a significantly increased incidence of the G0/G1 phase of cells following the hypoxia + pc-*SLC14A1* treatment, while that of the S phase decreased markedly compared to the hypoxia-alone treatment (*P* < 0.05), indicating that A498 cells were arrested in G1/S checkpoint under *SLC14A1* overexpression. The cell cycle distribution showed no significant changes between the hypoxia-alone group and the hypoxia + pc-NC group (*P* > 0.05, Fig. 4b). The hypoxia-alone treatment induced the invasion and migration of cells, but these changes were markedly abolished when combined with the pc-*SLC14A1* treatment (hypoxia + pc-SLC14A1 group), and the change was significant (*P* < 0.05). There was no significant change in the invasion and migration of A498 cells between the hypoxia-alone treatment and hypoxia + pc-NC treatment group (*P* > 0.05, Fig. 4c-d). Next, we determined the role of *SLC14A1* gene expression in inflammatory cytokine production in A498 cells. The hypoxia-induced elevations of IL-6 and TNF-α levels observed in the hypoxia-alone group were suppressed by the pc-*SLC14A1* treatment in the hypoxia + pc-*SLC14A1* group (*P* < 0.05). The IL-6 and TNF-α levels showed no significant differences between the hypoxia-alone group and the hypoxia + pc-NC group (*P* > 0.05, Fig. 4e-f).

## Discussion

Hypoxia, a typical microenvironment characteristic, leads to tumor development and metastasis. In the hypoxic microenvironment, cytokines are secreted in primary tumor cells and macrophages accumulate under altered gene expression [22]. The imbalance between oxygen consumption and oxygen supply is a crucial event at all stages of cancer development and is commonly associated with molecular and cellular characteristics of cancer, including mutagenesis and impaired DNA repair, resistance to radio- and chemo-therapy, EMT, and cancer metabolic reprogramming [23–25]. Hypoxia escalates tumor growth, inflammation, and immunosuppression, thereby transforming the tumor from benign to invasive [26]. Recent studies have profiled the related hypoxia- and immune system-related gene expression changes, which may be used to optimize prognostic risk stratification and individual therapy in triple-negative breast cancer patients [27]. Another study indicated that the hypoxia-induced RNA *miR-3677-3p* enhanced the proliferation, migration, and invasion of hepatocellular carcinoma cells by inhibiting sirtuin 5 [28]. Moreover, the exosome-mediated transfer of miR-193a-3p, miR-210-3p, and miR-5100 (which are derived from hypoxic bone marrow-derived mesenchymal stem cells) has been shown to induce lung cancer cell invasion by modulating signal transducer and activator of transcription 3 (STAT3) signaling pathway [29].

Our results are consistent with those obtained by the previous literature, namely that hypoxia promotes RCC cell viability, invasion, and migration in a time-dependent manner, while inhibiting RCC cell apoptosis. It is a generally accepted concept that hypoxia can induce inflammation, and the findings herein indeed indicate that hypoxia significantly induced the release of the serum inflammatory cytokines IL-6 and TNF-α in RCC cells. Thus, tumor hypoxia is a primary deleterious factor involved in malignant progression and hence represents a viable target for cancer treatment. There is convincing evidence to indicate that well-oxygenated cells (approximately 20–21% oxygen, 160 mmHg) are more sensitive to chemotherapy, radiotherapy, and immunotherapy; whereas hypoxic cells (approximately 2.6% oxygen, 20 mmHg) readily develop drug resistance and lead to poor treatment response [30]. Our research explored the effect of oxygen inhalation on the clinical efficacy of RCC patients. The results indicate that the oxygen administration intervention group had significantly decreased values of renal function indexes, serum inflammatory factors, and tumor markers. Moreover, the oxygen administration group had fewer postoperative complications and lower incidence rates of RCC metastasis and recurrence. As a result, the intervention of oxygen administration significantly alleviated the anxiety and depression of RCC patients, while nursing satisfaction was significantly improved. The above data suggest that nursing intervention based on oxygen administration can improve the clinical efficacy of RCC therapies, comprising a safe and effective nursing intervention method.

Our data show that hypoxia-regulated UT-B expression in cultured A498 cells significantly lower than that of the normoxia, so its encoding gene, SLC14A1, may be closely associated with hypoxia-induced RCC development, and the underlying mechanism is worth pursuing. The *SLC14A1* gene is located on chromosome 18q12.1–21.1 and encodes the human UT-B protein [31], a membrane channel protein that plays a crucial role in the passive transport of urea [32]. It was recently reported that the *SLC14A1* gene is associated with human malignancies, exhibiting downregulated expression in lung adenocarcinoma specimens, while its elevated expression was found to inhibit lung squamous cell carcinoma colony formation [33]. Another study similarly indicated that *SLC14A1* expression in malignant prostate cancer tissues was significantly low, being inversely proportional to both the clinical grade and stage [34]. It has been reported that several single nucleotide polymorphisms (SNPs) within the *SLC14A1* gene (such as rs1058396, rs2298720, rs11877062, and rs17674580) are strongly correlated with bladder cancer [35–38]. The Gene Expression Profiling Interactive Analysis (GEPIA) database shows that *SLC14A1* gene expression levels in KICH, KIRC, and KIRP tissues are significantly lower than that in corresponding normal renal tissue. A Kaplan–Meier plot of data obtained from Fig. 2d indicated that high *SLC14A1* gene expression prolongs overall survival to some extent in patients with RCC. Our experiments also demonstrated that enhanced *SLC14A1* gene expression induced mitochondrial dysfunction, including increased mitochondrial ROS generation, diminished intracellular ATP level, and the destruction of both *ΔΨm* integrity and mitochondrial morphology. Therefore, the *SLC14A1* gene appears to participate in the development and occurrence of RCC by regulating mitochondrial function.

Recent studies have explored the mechanisms and pathways of hypoxia in tumor progression and metastasis, such as canonical HIF signaling, mitochondrial function, the unfolded protein response (UPR), and methylation changes [39, 40]. ROS generation is responsible for causing progressive mitochondrial dysfunction and cell apoptosis [41, 42]. In our study, mitochondrial ROS accumulation was significantly enhanced in A498 cells transfected with the overexpression plasmid pc-*SLC14A1*; the toxic effect of ROS generation may be a crucial cause of RCC cell apoptosis. Moreover, the present study further confirmed that *SLC14A1* overexpression abolished the hypoxia-induced invasion and migration of A498 cells, and moreover, the cell cycle was arrested at the G1/S checkpoint. In addition, *SLC14A1* overexpression enhanced ROS accumulation, which in turn triggered A498 cell apoptosis. Hypoxia-induced elevations in the levels of IL-6 and TNF-α were suppressed by *SLC14A1* overexpression via a mitochondria-dependent pathway.

## Conclusion

These findings indicate that oxygen administration to patients does transiently relieve tumor hypoxia, and as a result, improves therapeutic outcomes. Our findings show that hypoxia induces changes in gene expression, such as the inhibition of *SLC14A1* gene expression, which has many important downstream cellular and physiological effects in RCC. Accordingly, enhancing the expression of the *SLC14A1* gene led to mitochondrial ROS accumulation, *ΔΨm* disruption, and abnormal mitochondrial morphology, which could abolish the hypoxia-induced invasion and migration of A498 cells and inhibit the hypoxia-induced release of inflammatory cytokines. These findings support a mechanism wherein the *SLC14A1* gene plays a crucial role in hypoxia-induced RCC in a mitochondria-dependent manner.

Our experimental work showed that *SLC14A1* gene expression is an important factor involved in hypoxia-induced RCC development and progression, but evidence from large cohort studies on its expression and association with clinical outcomes is lacking. Further research is therefore required to accurately analyze the effects of *SLC14A1* gene expression on each aspect of tumor progression (including proliferation, invasion, angiogenesis, and metastasis) to guide novel therapeutic strategies. Of note, the specific molecular mechanism by which hypoxia regulates the *SLC14A1* transcription level is still elusive and merits further investigation. Existing studies on therapeutic strategies for RCC have obvious limitations, such as small sample sizes, further research will be required to collect data from a larger RCC cohort. A therapeutic strategy for RCC should be supported by high-quality, evidence-based medicine, in the future, a large-scale, multicenter, randomized controlled clinical trial must be conducted to verify the effectiveness of oxygen administration to RCC patients. We will share and recommend this strategy in medical practice guidelines on a global platform to ensure future guidelines adhere to the principles of evidence-based medicine.

The development of specific agents for targeting hypoxia and its related pathways will also be crucial. These will have the potential to provide innovative cancer therapies that can enhance antitumor immunity and overcome the barriers of treatment resistance, tumor tolerance, and escape from immune surveillance. There exists the question of whether it will be possible to use therapeutic targets derived from the hypoxic tumor microenvironment and its related pathways as new therapeutic schemes for cancer immunotherapy, this deserves further investigation. Integrating the manipulation of hypoxic stress into cancer immunotherapy may lead to more durable and effective cancer immunotherapy approaches in the future.

## Methods

### Patient recruitment

The present study was based on the principles of the Declaration of Helsinki. This study was approved by the Medical Ethics Committee of Nanjing Hospital Affiliated to Nanjing Medical University (Date: 2016.11.17/No. 2016–227). The written informed consent of each participant was obtained. A total of 64 patients with RCC diagnosed at Nanjing Hospital Affiliated to Nanjing Medical University between Jan 2017 and Jan 2020 were recruited into the study cohort. Among them, there were 32 cases in the control group (15 females and 17 males), in which, the average age was 52.1 years old, and the histological type was as follows: 5 cases of KICH, 19 cases of KIRC, and 8 cases of KIRP. There were 32 cases in the observation group (18 females and 14 males), in which, the average age was 51.9 years old, and the histological type was as follows: 4 cases of KICH, 21 cases of KIRC, and 7 cases of KIRP. The inclusion criteria were as follows: ① RCC histological diagnosis was based on both hematoxylin and eosin (H&E) staining and immunochemistry results; ② the clinical data of patients were complete; ③ the patients underwent radical nephrectomy. The exclusion criteria were as follows: ① patients with other malignant tumors; ② patients with severe postoperative complications caused by uncontrollable basic diseases; ③ patients with incomplete clinical case data. There was no significant difference in patients’ clinical and pathological characteristics between these two groups.

### Nursing intervention

The control group patients received conventional nursing care, including as preoperative psychological intervention, postoperative anti-infection, complication care, and rehabilitation training. Using conventional nursing care as a reference, the observation group (evidence-based nursing model group) implemented the evidence-based nursing model for the patients. Namely, the PubMed, EMBASE, and Cochrane Library databases were searched for entries from Jan 2010 to Jan 2020 for literature regarding difficulties in clinical practice following RCC operation, and a systematic evaluation and meta-analysis were employed to guide the nursing model for RCC postoperative complications. From this, an evidence-based nursing model was developed and used in the present study, which employed the following: ①oxygen inhalation, involving monitoring the blood oxygen saturation (> 90%), oxygenation index (> 300 mmHg), blood lactic acid concentration, and evaluating the degree of hypoxia in patients; ② local oxygen therapy, whereby the surgical wound was treated with local oxygen therapy for 90 min/d for 5 consecutive days and 2 intermittent days, while the wound pressure was maintained at 3 kPa during the care process; ③ complications during the nursing intervention: a common complication after RCC operation is anastomotic leakage with abdominal infection, and an evidence-based nursing model must judge the occurrence of anastomotic bleeding and abdominal leakage according to the patient's abdominal pain, abdominal distension, blood pressure, body temperature, and drainage fluid characteristics.

### Clinical index observation

The venous blood from RCC patients was collected. Samples from patients with jaundice, hemolysis, and high lipids were not included. Renal function indexes (BUN and Cr) and serum inflammatory factors (IL-6 and TNF-α) were detected by the Beckman Coulter AU5800 automatic biochemical analyzer. Tumor markers (CEA and CA50) were measured by the Roche Cobas e601 automatic electrochemiluminescence immunoanalyzer. The Self-Rating Anxiety Scale (SAS, > 50 for anxiety) and Self-Rating Depression Scale (SDS, > 53 for depression) were used to evaluate the psychological status of RCC patients. Nursing satisfaction was scored as follows: satisfied, 4 points; somewhat satisfied, 3 points; dissatisfied, 2 points; very dissatisfied, 1 point. The nursing satisfaction rate was calculated as the sum of the given answers ‘satisfied’ and ‘somewhat satisfied’ over the total number of responses.

### Postoperative follow-up

RCC patients were rechecked every 3 months after their operation, whereby each underwent a routine blood test, routine urine test, biochemical test, chest radiograph, and abdominal and urinary ultrasound test. If there was suspicion of RCC recurrence, urinary computed tomography (CT), chest CT, and a bone scan were conducted.

### Cell culture

The human RCC cell line A498 was obtained from the Cell Bank of China Union Medical University (Beijing, China). A498 cells were grown in Dulbecco’s modified Eagle’s medium (DMEM, CAT#: 01–050-1A, Life Technologies, Grand Island, NY, USA) supplemented with 10% heat-inactivated fetal bovine serum (FBS, CAT#: 04–127-1A, Life Technologies, Grand Island, NY, USA) and 100 μg/ml penicillin/streptomycin (CAT#: PSF-1, Life Technologies, Grand Island, NY, USA), then maintained at 37 °C in constant 5% CO_2_ and 90–100% humidified atmosphere. The A498 cells underwent annual short tandem Repeat (STR) profiling analysis, population doubling time and morphology for genetic confirmation. A498 cells in the logarithmic growth phase were divided into two groups: ① the hypoxic condition group at 37 °C, 5% CO_2_, 2% O_2_, and 93% N_2_; ② the normoxic condition group at 37 °C, 5% CO_2_, and 95% O_2_. All experiments were performed with early-passage cells (passages 4–10).

### SLC14A1 plasmid construction and transfection.

*SLC14A1* cDNA was subcloned into a pcDNA3.1 mammalian expression vector (CAT#: V79520, Invitrogen Life Technologies, Carlsbad, CA, USA). The primer sequences of the pcDNA3.1-*SLC14A1* (pc-*SLC14A1*) plasmid were as follows: *SLC14A1*-sense, 5'-CCA GTG GGA GTT GGT CAG AT-3'; *SLC14A1*-antisense, 5'-GTT GAA ACC CCA GAG TCC AA-3′. The reconstituted pc-*SLC14A1* plasmid and empty plasmid (pc-NC) were transfected into A498 cells. Briefly, 2.5 μg of either the pc-*SLC14A1* plasmid or pc-NC plasmid was mixed with 8 μL of lipofectamine 2000 (CAT#: 11,668,019, Invitrogen Life Technologies, Carlsbad, CA, USA) and incubated with A498 cells for 6 h. The cells were then cultured in DMEM containing 20% FBS for another 48 h.

### Western blot analysis

A498 cells were harvested and lysed in radioimmunoprecipitation assay (RIPA) buffer. Equal amounts of cellular protein were loaded per lane, subjected to 12% sodium dodecyl sulfate–polyacrylamide gel electrophoresis (SDS-PAGE), and subsequently transferred to a polyvinyl difluoride (PVDF) membrane (CAT#: GVWP02500, Millipore, Billerica, MA, USA). The membrane was then blocked with 5% skimmed milk for 1 h and immunoblotted with primary antibodies specific to SLC14A1 (1: 100 dilution, CAT#: AV48116; Sigma-Aldrich, St. Louis, MO, USA) and actin (1: 5,000; CAT#: A5441; Sigma-Aldrich, St. Louis, MO, USA) at 4 °C overnight. The blot was then incubated with goat anti-rabbit IgG horseradish peroxidase-conjugated secondary antibody (1: 5,000; CAT#: SA00001-2; ProteinTech Group, Chicago, IL, USA). An enhanced chemiluminescence (ECL) western detection system (CAT#: 7003; Cell Signaling Technology, Beverly, MA, USA) was used to detect immunoreactive bands.

### Electron microscopy

A498 cells were harvested in 1 mm^3^ blocks and immersed in 3% glutaraldehyde solution for 2 h. The samples were post-fixed in 1% osmium tetroxide (OsO_4_; CAT#: 18,459; Ted Pella, Redding, CA, USA) for 2 h, dehydrated in ascending alcohols, and then embedded in Eponate 12 resin (CAT#: 18,005; Ted Pella, Redding, CA, USA). Ultrathin (60 nm) sections were cut and post-stained with 5% uranyl acetate (CAT#: 19,481; Ted Pella, Redding, CA, USA) and lead citrate (CAT#: 19,312; Ted Pella, Redding, CA, USA). The mitochondrial ultrastructure of A498 cells was observed under a transmission electron microscope (JEM 1011, Japan).

### Measurement of mitochondrial ROS generation

The mitochondrial ROS level in A498 cells was analyzed by the MitoSOX™ Red mitochondrial superoxide indicator. In brief, A498 cells were subjected to different treatments and incubated with MitoSOX™ Red (5 µM, CAT#: M36008, Invitrogen Life Technologies, Carlsbad, CA, USA) in the dark at 37 °C for 20 min. The fluorescence intensity was evaluated using a flow cytometer (BD FACSCalibur).

### Determination of ΔΨm

Changes in the *ΔΨm* of A498 cells were measured using the fluorescent cationic dye 5,5′,6,6′-tetrachloro-1,1′,3,3′-tetraethyl-imidacarbocyanine iodide (JC-1). Briefly, A498 cells were subjected to various treatments and then incubated with JC‑1 (20 µg/mL, CAT#: T3168, Invitrogen Life Technologies, Carlsbad, CA, USA) in the dark at 37 °C for 15 min. The fluorescence intensity was evaluated using an epifluorescence microscope (Nikon Corp., Tokyo, Japan).

### Measurement of ATP level

A luciferase-based assay kit (CAT#: S0026; Beyotime, Shanghai, China) was used to determine the intracellular ATP content. A498 cells were lysed with ATP lysis buffer and centrifuged at 12,000 g for 8 min at 4 °C. The resulting supernatant and ATP-detection working solution were then mixed before luminescence was measured using a BioTek Synergy H^1^ Microplate Reader (BioTek Instrument Inc., Winooski, VT, USA). The ATP level was determined according to the standard curve.

### Cell viability assay

The Cell Counting Kit-8 (CCK-8; CAT#: CK04; Dojindo, Kyushu Island, Japan) was employed to evaluate A498 cell viability. Briefly, A498 cells underwent various treatments before being incubated with CCK-8 solution (10 μL/100 μL) at 37 °C for 2 h. The absorbance was then assessed using a BioTek Synergy H^1^ Microplate Reader (BioTek Instrument Inc., Winooski, VT, USA) at 450 nm.

### Cell apoptosis and cell cycle assay

Annexin V-FITC/PI staining (CAT#: A211-01/02; Vazyme Biotech Co., Ltd., Nanjing, China) was used to evaluate A498 cell apoptosis. A498 cells underwent various treatments before being harvested and resuspended with 500 μL of binding buffer. Subsequently, the A498 cells were stained with 5 μL of Annexin V and 10 μL of PI (CAT#: A211-01/02; Vazyme Biotech Co., Ltd., Nanjing, China) for 30 min in the dark. Then, apoptotic cells were detected using a flow cytometer (BD FACSCalibur).

### Cell cycle assay

A498 cells underwent various treatments before being harvested and fixed in 70% ethanol for 18 h. The A498 cells were then stained with 50 mg/mL PI (CAT#: A211-01/02; Vazyme Biotech Co., Ltd., Nanjing, China) for 30 min at 37 °C. Finally, the cell cycle distribution was determined using a flow cytometer (BD FACSCalibur).

### Cell invasion assay

A transwell chamber (CAT#: CLS3422; Corning Incorporated, Corning, NY, USA) was employed to assess the invasive ability of A498 cells. In brief, starved A498 cells (5 × 10^6^/mL) were seeded into the top chamber (pore size, 8 μm), while 500 μL of DMEM containing 20% FBS was filled into the lower chamber. Following incubation for the indicated time, the invasive cells were fixed and stained with 0.1% crystal violet (CAT#: C8470; Solarbio Science Technology Co., Ltd., Beijing, China). The invasive ability of A498 cells was then evaluated under the microscope (× 200 magnification, Olympus, Tokyo, Japan).

### Cell migration assay

The wound healing assay was used to estimate the migratory ability of A498 cells. In brief, A498 cells were passaged to attain 80–90% confluence and were then scratched in a straight line on the surface of the monolayer cells using a 200 µl tip (at time point 0). After incubation durations of 24 h and 48 h, the healing width of the wound was surveyed with an inverted optical microscope (× 200 magnification, Olympus, Tokyo, Japan).

### Statistical analysis

Statistical analysis was conducted using SPSS software version 13.0 (SPSS Inc. Chicago, IL, USA). The data were presented as the mean ± standard deviation (SD). Comparisons between groups were conducted the Student's* t*-test if the data were normally distributed, otherwise, the Wilcoxon rank-sum test was performed. *P* < 0.05 was set to indicate statistical significance.

## Supplementary Information

**Additional file 1: Supplementary Figures** 

## Data Availability

The datasets generated during and/or analyzed during the current study are available from the corresponding author on reasonable request.

## Abbreviations

RCC: Renal cell carcinoma
ccRCC: Clear cell RCC
EMT: Epithelial-to-mesenchymal transition
VEGF: Vascular endothelial growth factor
GLUT1: Glucose transporter isoform 1
HIF: Hypoxia-inducible transcription factor
PI3K: Phosphatidylinositol 3-kinase
mTOR: Mammalian target of rapamycin
ROS: Reactive oxygen species
UT‐B: Urea transporter B
SLC14A1: Solute carrier family 14 member 1
BUN: Urea nitrogen
Cr: Creatinine
IL-6: Interleukin 6
TNF-α: Tumor necrosis factor α
CEA: Carcinoembryonic antigen
CA50: Carbohydrate antigen 50
KICH: Kidney Chromophobe
KIRC: Kidney renal clear cell carcinoma
KIRP: Kidney renal papillary cell carcinoma
HR: Hazard ratio
ΔΨm: Mitochondrial membrane potential
ATP: Adenosine triphosphate
PI: Propidium iodide
STAT3: Signal transducer and activator of transcription 3
SNPs: Single nucleotide polymorphisms
GEPIA: Gene Expression Profiling Interactive Analysis
UPR: Unfolded protein response
H&E: Hematoxylin and eosin
SAS: Self rating Anxiety Cale
SDS: Self rating Depression Scale
CT: Computed tomography
DMEM: Dulbecco’s modified Eagle’s medium
FBS: Fetal bovine serum
RIPA: Radio immuno precipitation assay
SDS-PAGE: Sodium dodecyl sulfate–polyacrylamide gel electrophoresis
PVDF: Polyvinyl difluoride
ECL: Enhanced chemiluminescence
OsO4: Osmium tetroxide
JC-1: Fluorescent cationic dye 5, 5′, 6, 6′-tetrachloro-1, 1′, 3, 3′-tetraethyl-imidacarbocyanine iodide
CCK-8: Cells Counting Kit-8
FITC: Fluorescein isothiocyanate
SD: Standard deviation
GTEx: Genotype-Tissue Expression
STR: Short tandem Repeat
