# Supplementary material for: Role of the human solute carrier family 14 member 1 gene in hypoxia-induced renal cell carcinoma occurrence and its enlightenment to cancer nursing
Source: BMC Mol Cell Biol. 2023 Mar 18;24:10. doi: 10.1186/s12860-023-00473-6 (PMC10024409; doi:10.1186/s12860-023-00473-6)
Supplement: Supplementary file 1 — Additional file 1: Supplementary Figures [file 12860_2023_473_MOESM1_ESM.docx]

**Supplementary figure 1. Certificate of Language Editing.**


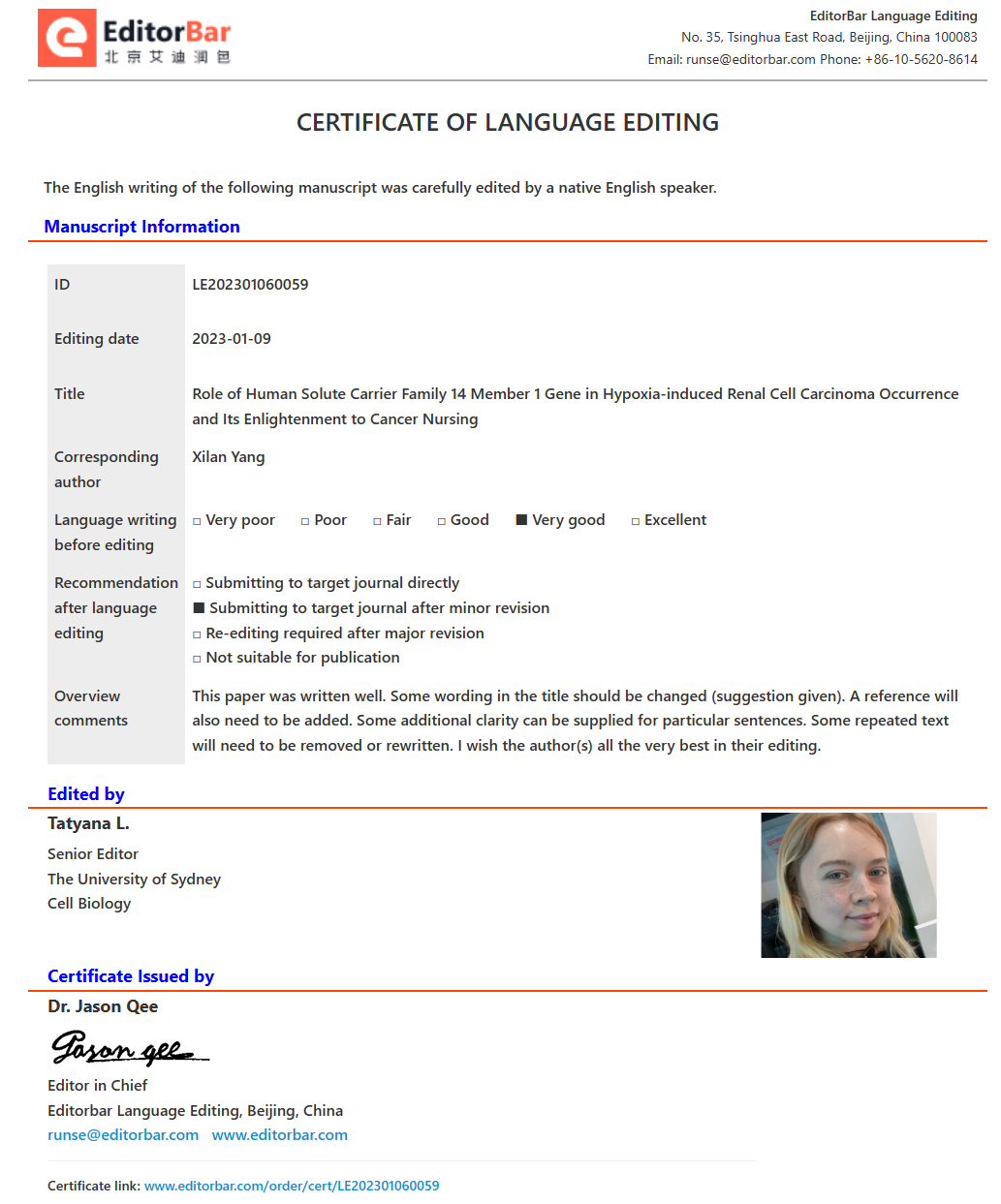


**Supplementary figure 2. Graphical abstract.**

**
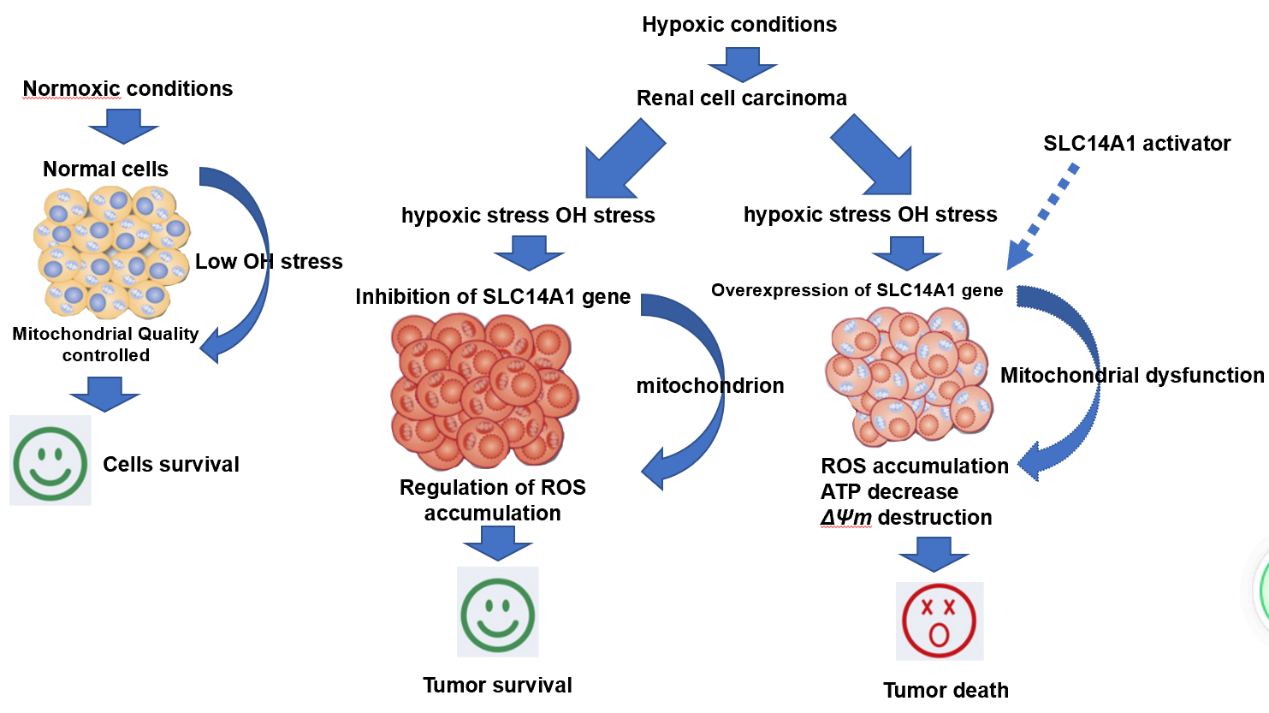
**

Supplementary figure 2. Hypoxic stress is considered a critical contributor to renal cell carcinoma growth and progression, including intra-tumor heterogeneity and the emergence of cancer clones resistant to therapies and anti-tumor immunity.

**Supplementary figure 3. Clinicopathological features of renal cell carcinoma patients.**

| **Clinicopathologic features** | **Cases (*n*)** | |
| --- | --- | --- |
|  |  |  |
| Gender | |  |
| male | | 31 |
| female | | 33 |
| Age (years) | |  |
| <60 | | 34 |
| ≥60 | | 30 |
| Histological types | |  |
| KICH | | 9 |
| KIRC | | 40 |
| KIRP | | 15 |
| Clinical stages | |  |
| Ⅰ~Ⅱ stages | | 22 |
| Ⅲ~Ⅳ stages | | 42 |
| Histological grading | |  |
| Ⅰ~Ⅱ grade | | 47 |
| Ⅲ~Ⅳ grade | | 17 |
| Paraneoplastic syndrome | |  |
| Yes | | 12 |
| No | | 52 |
| Distant metastasis | |  |
| Yes | | 10 |
| No | | 54 |
| Tumor thrombus in inferior vena cava | |  |
| Yes | | 18 |
| No | | 46 |

**Supplementary figure 4. Informed consent.**


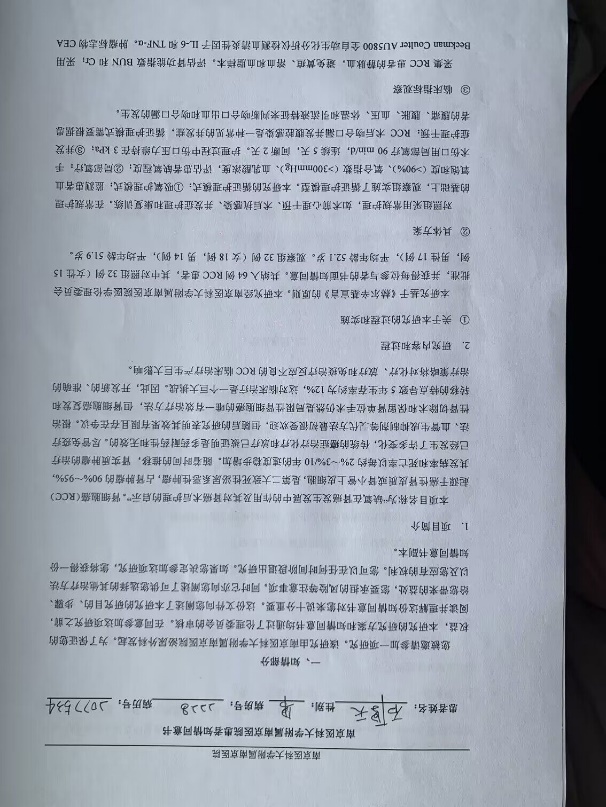

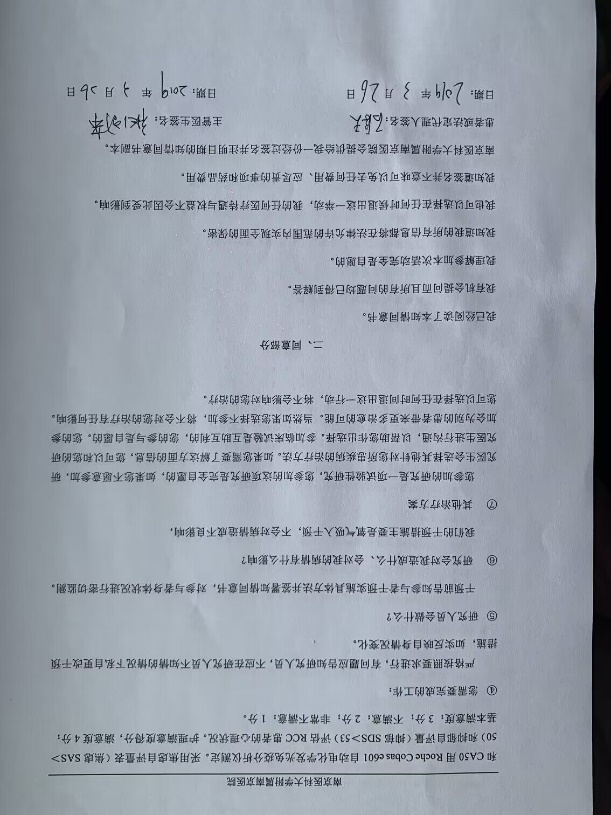


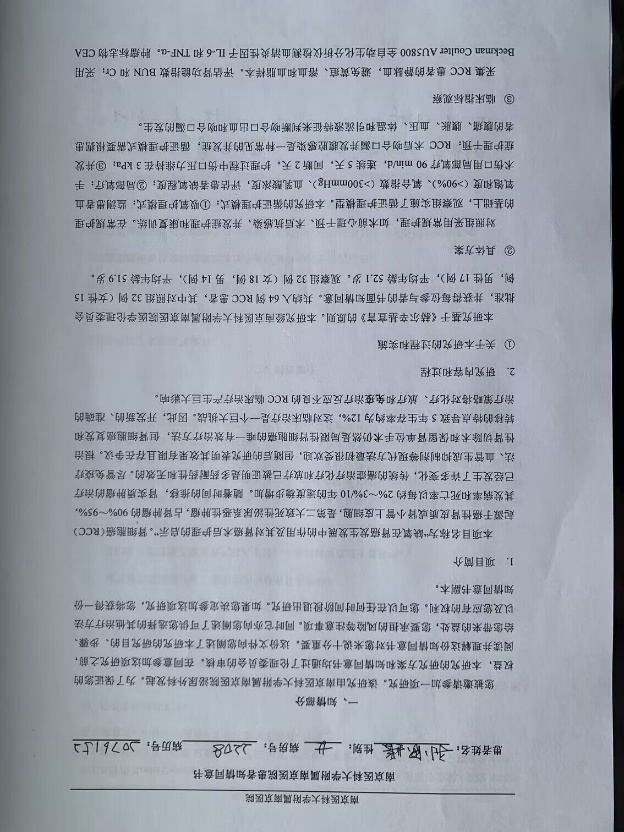

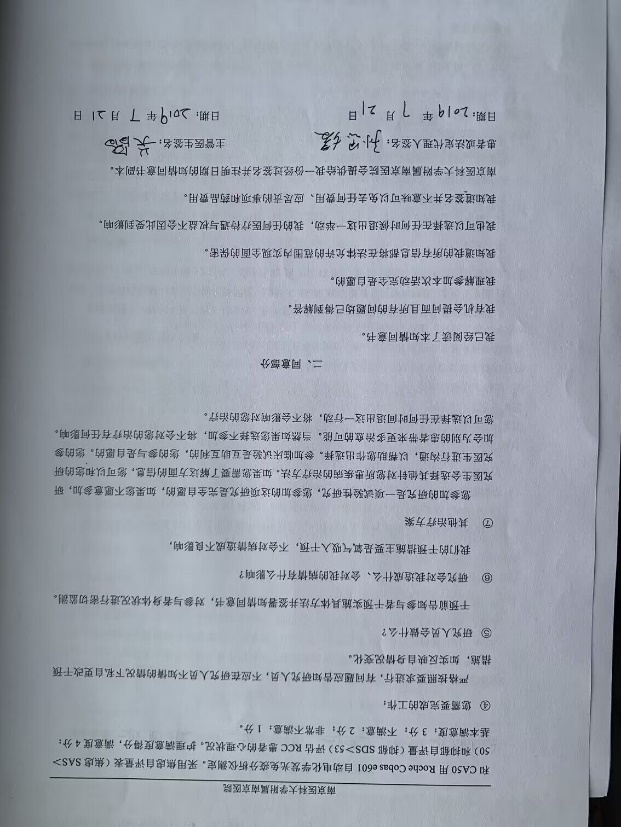


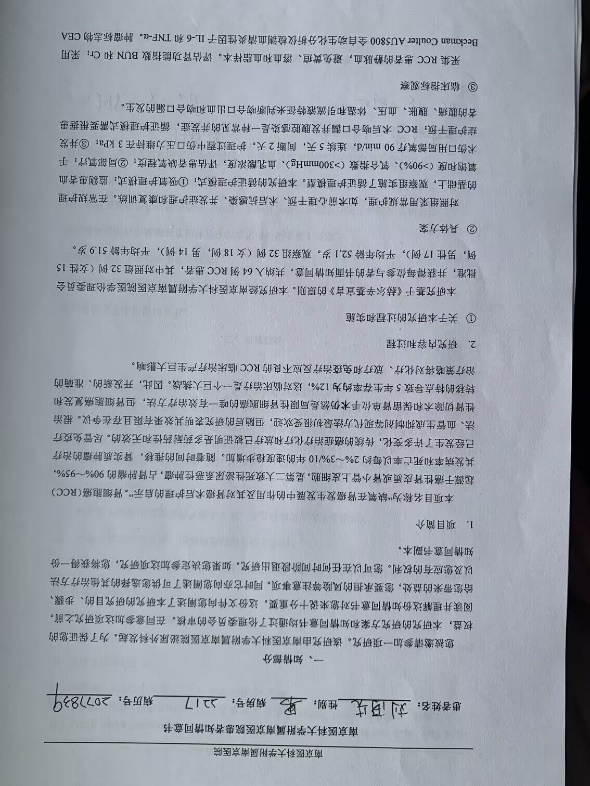

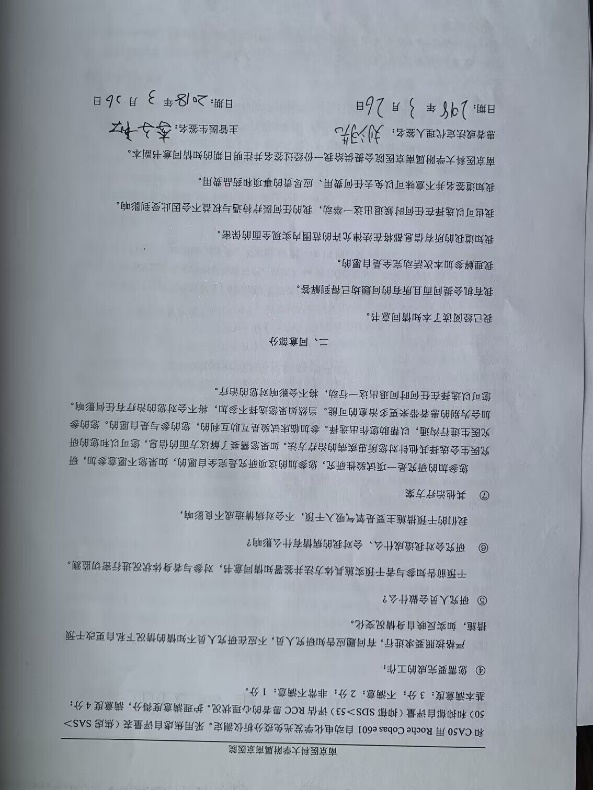


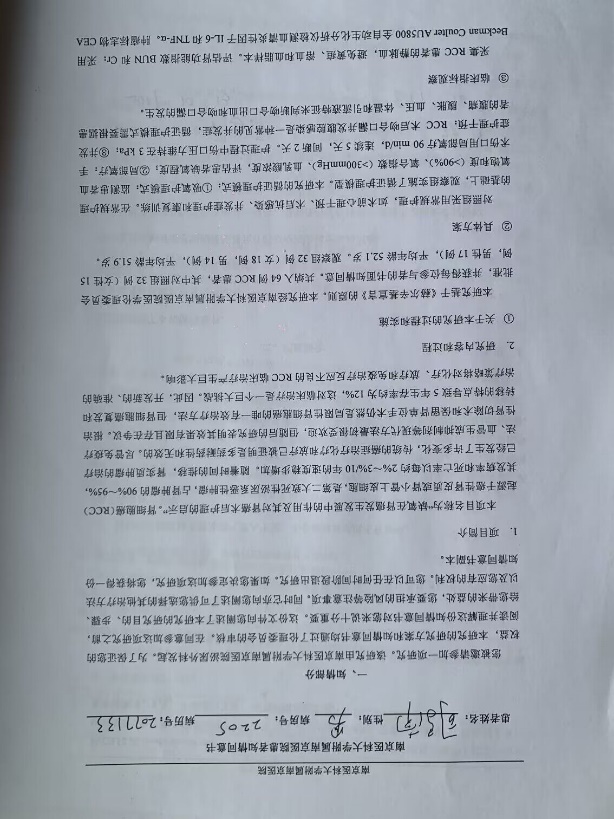

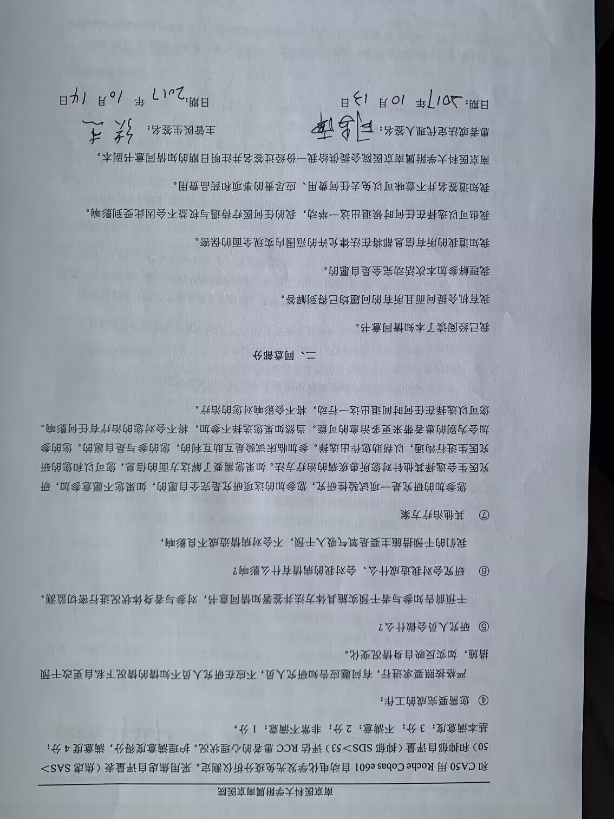


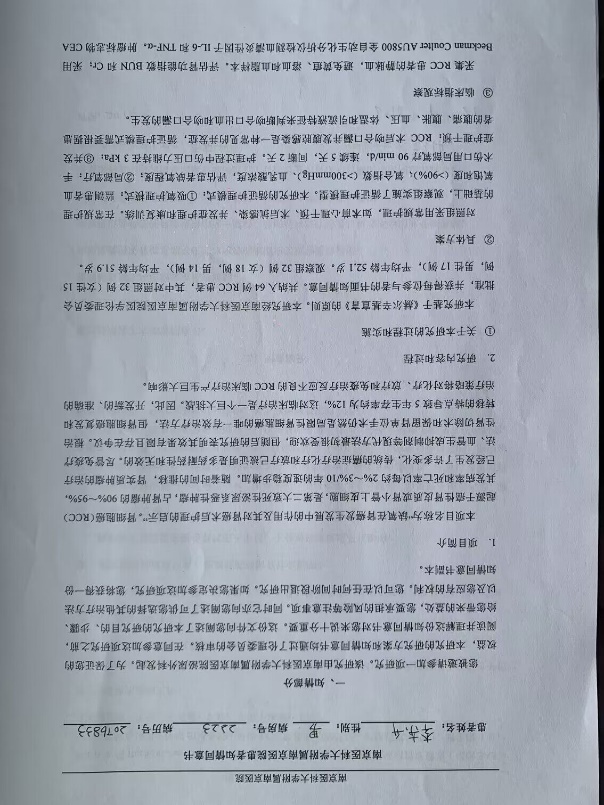

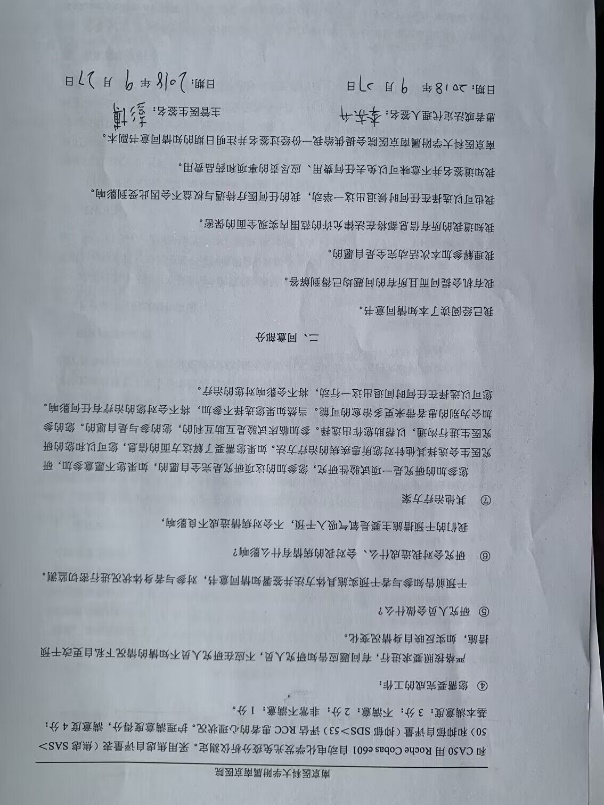


**Supplementary figure 5. Uncropped western blot images.**


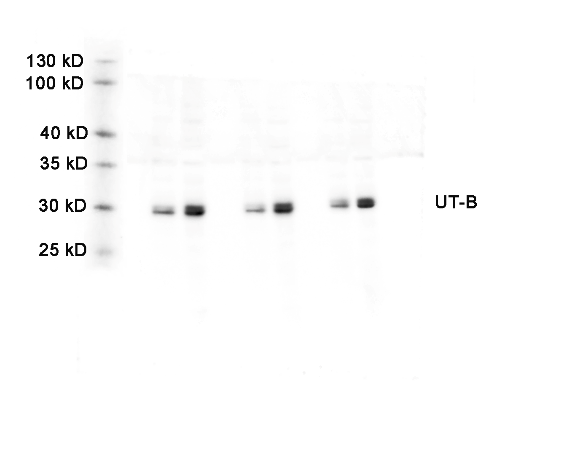

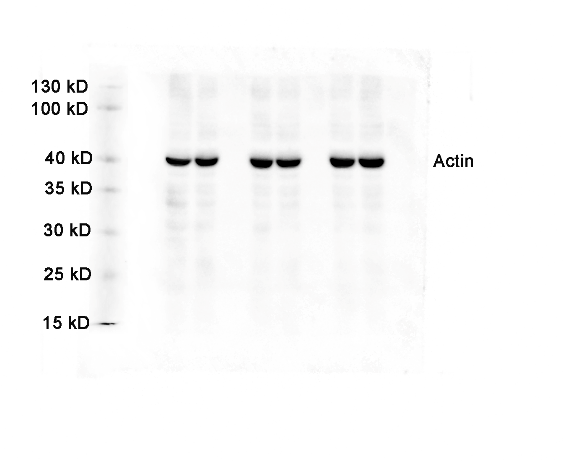


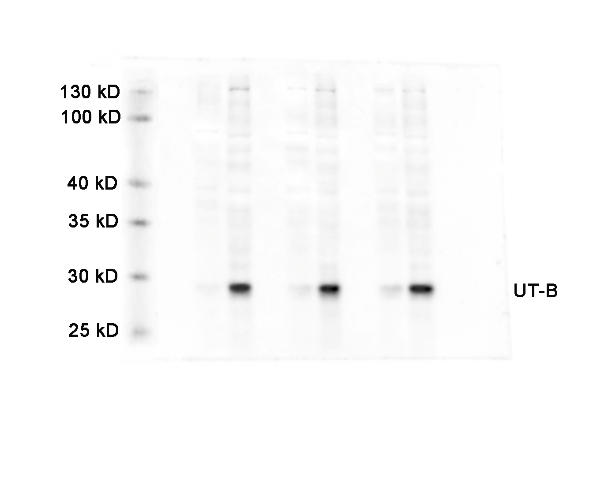

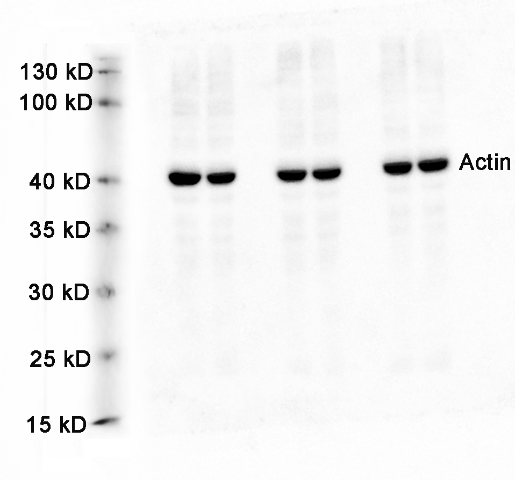


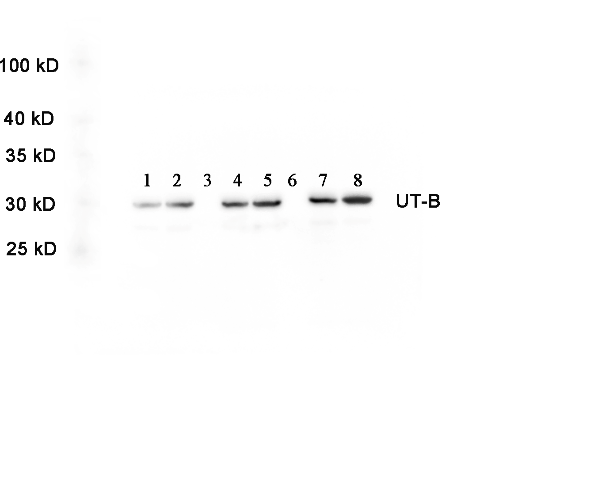

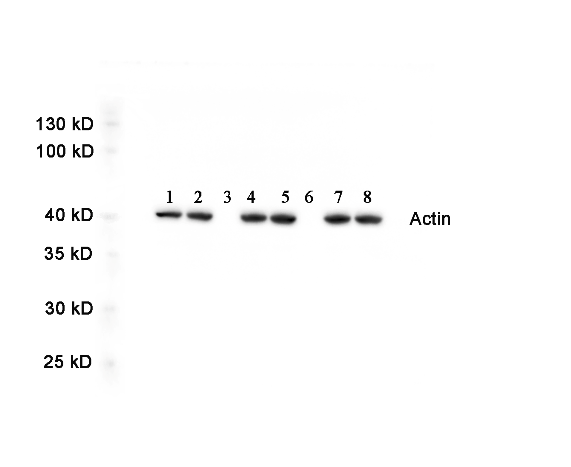


Supplementary figure 5. Effect of hypoxia on the biological characteristics of A498 cells. A498 cells were cultured at 37°C in a humidified incubator with 5% CO_2_, 2% O_2_, and 93% N_2_ (hypoxia) or with 5% CO_2_ and 95% O_2_ (normoxia) for 24 h and 48 h. The level of the UT-B and actin was determined by western blot. Full-length blots/gels are presented. Lanes 1, 2, 4, 5, 7 and 8 represent the western blot analysis of the UT-B and actin protein.

**Supplementary figure 6. Effect of hypoxia/normoxia on the invasion of A498 cells.**

**0 h 24 h 48 h**


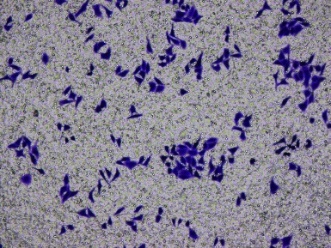

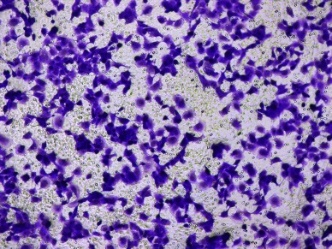

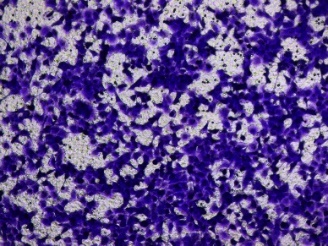
 **Hypoxia**


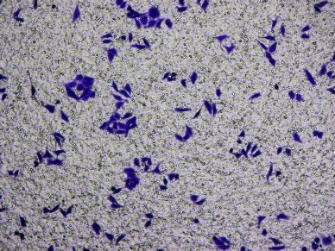

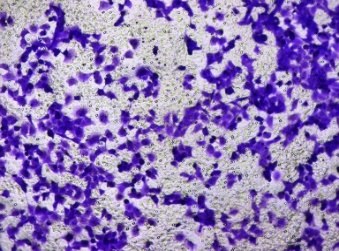

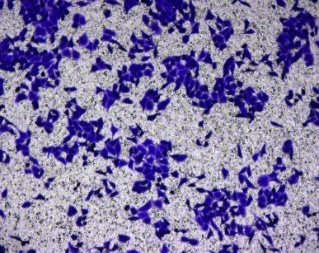
 **Normoxia**

Supplementary figure 6. A498 cells were cultured at 37°C in a humidified incubator with 5% CO_2_, 2% O_2_, and 93% N_2_ (hypoxia) or with 5% CO_2_ and 95% O_2_ (normoxia) for 24 and 48 h. The invasion of A498 cells was detected by the transwell assay.

**Supplementary figure 7. Effect of hypoxia/normoxia on the** **migration of A498 cells.**

**0 h 24 h 48 h**

**
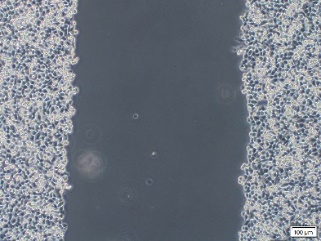

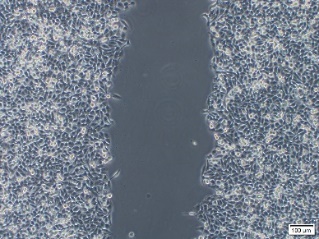

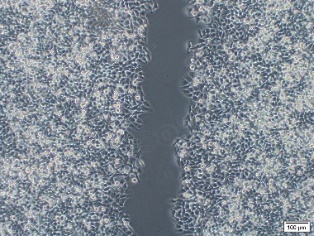
 Hypoxia**


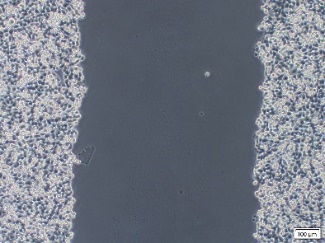

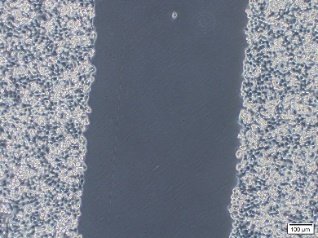

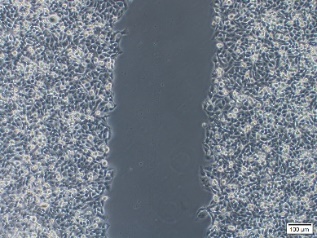
 **Normoxia**

Supplementary figure 7. A498 cells were cultured at 37°C in a humidified incubator with 5% CO_2_, 2% O_2_, and 93% N_2_ (hypoxia) or with 5% CO_2_ and 95% O_2_ (normoxia) for 24 and 48 h. The migration of A498 cells was examined by the wound healing assay.

**Supplementary figure 8. Effect of *SLC14A1* overexpression on mitochondrial dysfunction in A498 cells.**









**Mock pc-SLC14A1 pc-NC**

Supplementary figure 8. Observation of A498 cell morphology was performed using electron microscopy (×5,200). Scale bar = 1 μm.

**Supplementary figure 9. Effect of *SLC14A1* overexpression on hypoxia-induced migration of A498 cells.**


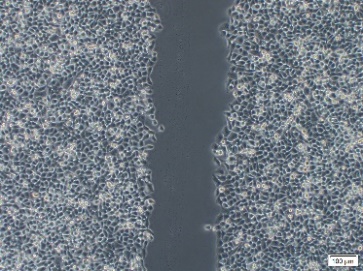

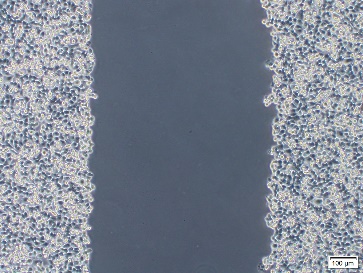

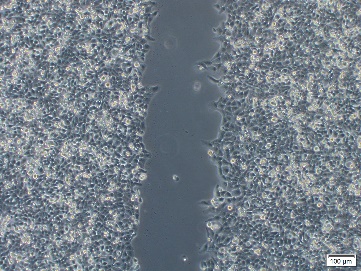


**hypoxia hypoxia+pc-SLC14A1 hypoxia+pc-NC**

Supplementary figure 9. A498 cells were transfected with either the pc-*SLC14A1* vector (2.5 μg/ml) or pcDNA3.1 null vector (pc-NC, 2.5 μg/ml) and then cultured at 37°C in a humidified incubator with 5% CO_2_, 2% O_2_, and 93% N_2_ (hypoxia) for 48 h. The migration of A498 cells was analyzed by the wound healing assay.
